# Long-term outcome of delirium during intensive care unit stay in survivors of critical illness: a prospective cohort study

**DOI:** 10.1186/cc13929

**Published:** 2014-06-18

**Authors:** Annemiek E Wolters, Diederik van Dijk, Wietze Pasma, Olaf L Cremer, Marjolein F Looije, Dylan W de Lange, Dieuwke S Veldhuijzen, Arjen JC Slooter

**Affiliations:** 1 Department of Intensive Care Medicine, University Medical Center Utrecht, Heidelberglaan 100, 3508 GA Utrecht, The Netherlands

## Abstract

**Introduction:**

Delirium is associated with impaired outcome, but it is unclear whether this relationship is limited to in-hospital outcomes and whether this relationship is independent of the severity of underlying conditions. The aim of this study was to investigate the association between delirium in the intensive care unit (ICU) and long-term mortality, self-reported health-related quality of life (HRQoL), and self-reported problems with cognitive functioning in survivors of critical illness, taking severity of illness at baseline and throughout ICU stay into account.

**Methods:**

A prospective cohort study was conducted. We included patients who survived an ICU stay of at least a day; exclusions were neurocritical care patients and patients who sustained deep sedation during the entire ICU stay. Delirium was assessed twice daily with the Confusion Assessment Method for the ICU (CAM-ICU) and additionally, patients who received haloperidol were considered delirious. Twelve months after ICU admission, data on mortality were obtained and HRQoL and cognitive functioning were measured with the European Quality of Life – Six dimensions self-classifier (EQ-6D). Regression analyses were used to assess the associations between delirium and the outcome measures adjusted for gender, type of admission, the Acute Physiology And Chronic Health Evaluation IV (APACHE IV) score, and the cumulative Sequential Organ Failure Assessment (SOFA) score throughout ICU stay.

**Results:**

Of 1101 survivors of critical illness, 412 persons (37%) had been delirious during ICU stay, and 198 (18%) died within twelve months. When correcting for confounders, no significant association between delirium and long-term mortality was found (hazard ratio: 1.26; 95% confidence interval (CI) 0.93 to 1.71). In multivariable analysis, delirium was not associated with HRQoL either (regression coefficient: -0.04; 95% CI -0.10 to 0.01). Yet, delirium remained associated with mild and severe problems with cognitive functioning in multivariable analysis (odds ratios: 2.41; 95% CI 1.57 to 3.69 and 3.10; 95% CI 1.10 to 8.74, respectively).

**Conclusions:**

In this group of survivors of critical illness, delirium during ICU stay was not associated with long-term mortality or HRQoL after adjusting for confounding, including severity of illness throughout ICU stay. In contrast, delirium appears to be an independent risk factor for long-term self-reported problems with cognitive functioning.

## Introduction

Because of improved medical care, the number of intensive care unit (ICU) survivors has increased considerably, but recent studies demonstrate that ICU survivors can experience substantial long-term morbidity [1-5]. To further improve care for survivors of critical illness, it is important to elucidate which factors increase the risk of long-term morbidity and mortality. Delirium, characterized by an acute change in attention and cognition, is a common disorder in ICU patients [6-8]. Previous studies have consistently found that delirium in the ICU is associated with long-term mortality and cognitive impairment [9-14]. It is, however, unclear whether delirium also affects long-term health related quality of life (HRQoL). HRQoL is defined as health, in the medical definition, but also as the importance of independent physical, social and emotional functioning [15]. Two studies suggest that delirium is a risk factor for lower long-term HRQoL [12,14], while others state that there is no association [11,16].

However, the relationship of delirium with long-term outcome is complex and affected by several confounding factors [17]. Previous studies adjusted for severity of illness at baseline, but only one investigation additionally adjusted for severity of illness during the ICU stay [10], which is another potentially important determinant of long-term outcome [18]. In that study, delirium was found to increase the risk of long-term cognitive impairment, independent of the burden of disease during the entire ICU stay [10]. It is currently unclear to what extent the associations between delirium and long-term mortality and HRQoL are confounded by severity of illness throughout the course of the ICU stay.

We conducted a large prospective cohort study of a diverse population of ICU survivors to assess the association between delirium in the ICU and long-term mortality, self-reported HRQoL and self-reported problems with cognitive functioning, while adjusting for important confounding factors such as severity of illness at baseline and throughout the course of the ICU stay. We hypothesized that persons who were delirious during their ICU stay had worse long-term outcomes compared to individuals who were not delirious.

## Materials and methods

### Study design

We performed a prospective cohort study within a larger follow-up investigation of all patients admitted to the ICU of the University Medical Center Utrecht (UMCU) in the Netherlands. The Medical Research Ethics Committee of the UMCU approved this study and waived the need to ask for informed consent (protocol 10/006), since the objective of the follow-up investigation was continuous quality assessment and evaluation of regular patient care.

### Study population

We included all patients who were admitted to the ICU of the UMCU for more than 24 hours, between July 2009 and August 2011. We excluded neurotrauma, neurosurgery and neurology patients, because the sensitivity of the Confusion Assessment Method for the ICU (CAM-ICU) is less reliable in patients with neurological disorders [7]. In this investigation, we aimed to study mortality and morbidity in ICU survivors; therefore, we excluded all patients who died during their ICU stay. Additionally, we excluded patients who had a sustained Richmond Agitation Sedation Scale (RASS) of -4 or -5, as delirium screening could not be conducted in these patients. Furthermore, we excluded all subjects who had no data on Sequential Organ Failure Assessment (SOFA) scores.

To determine which subjects were alive one year after ICU admission, the hospital information system and the Dutch municipal database were consulted. Individuals who could not be traced were excluded. Non-survivors were defined as all persons known to have died after discharge from the ICU during the one-year follow-up period.

### Measurements during ICU stay

The Acute Physiology and Chronic Health Evaluation IV (APACHE IV) was registered for every patient in the ICU after the first 24 hours of admission as an estimate of baseline severity of illness. In addition, SOFA was scored three times daily for the entire ICU length of stay and the cumulative score was used to provide an estimate of severity of illness throughout the course of the ICU stay. To calculate the cumulative SOFA score, we summed all daily SOFA scores without the central nervous system component, as this is altered in delirium and otherwise analyses would be subject to overcorrection. The cumulative SOFA score was considered a better measure than the mean or maximum SOFA score as it better represents the burden of illness over the entire ICU stay. We conducted sensitivity analyse using the mean SOFA and maximal SOFA in the analysis of the primary outcome, mortality. The presence of delirium was assessed twice daily by bedside nurses using the CAM-ICU, in the morning and evening shift [19]. Further, the administration of haloperidol was considered. Delirium during the ICU stay was defined as at least one positive CAM-ICU and/or administration of haloperidol during the ICU stay. In our clinic, haloperidol was not prescribed prophylactically during this study and, therefore, could be used as an indicator of delirium [20].

### Outcomes

The primary outcome was mortality in survivors of critical illness within the first year after ICU admission, while adjusting for important confounding factors, such as severity of illness at baseline and throughout the course of their ICU stay. Secondary outcomes were self-reported HRQoL and self-reported problems with cognitive functioning, one year after ICU admission, adjusted for the same confounders. To assess mortality, we used all available information, including the hospital information system and the Dutch municipal database. Persons who survived up to one year after admission to the ICU received a postal questionnaire to assess their physical and psychological well-being. Phone calls were made to all persons who did not reply to the survey, to increase the response rate. Non-responders were all subjects who received a survey but did not return or did not fill in the survey. The questionnaire included the Dutch European Quality of Life – Six Dimensions self-classifier (EQ-6D), which is an instrument that measures quality of life in six dimensions. Every dimension consists of one item, subdivided into three levels (no, mild and severe) [21]. To assess HRQoL, we analyzed the first five questions of the Dutch EQ-6D which corresponds to the validated Dutch European Quality of Life – Five Dimensions self-classifier (EQ-5D^TM^) [21,22]. We used a validated syntax to calculate the EQ-5D^TM^ index [22-24]. For assessment of problems with cognitive functioning we used the sixth question of the EQ-6D questionnaire [21].

### Statistical analysis

For categorical data a Chi-square test was used. To assess whether or not continuous data were normally distributed, a Q-Q plot was made and a Kolmogorov-Smirnov test was conducted. For normally distributed, continuous data, a Student T-test was used, and values presented as means and standard deviations (SD). Skewed, continuous data were studied with the Mann–Whitney U test and presented as medians with interquartile ranges (IQR).

To study the association between delirium and mortality, Cox proportional hazard regression analyses were used, and a hazard ratio (HR) was computed. HRQoL was compared between persons who had been delirious and those who had not been delirious during their ICU stay, using multivariable linear regression, which is presented as a regression coefficient. The HRQoL in these two groups was also compared with the HRQoL normative scores of the general Dutch population. Problems with cognitive functioning were subdivided into no problems, mild problems and severe problems. Multinomial logistic regression was performed to quantify the association between delirium and problems with cognitive functioning, and data are presented as odds ratio (OR).

In the analyses on mortality, HRQoL, and problems with cognitive functioning, the following two models were applied: 1. Model 1 did not contain additional covariates and was used for unadjusted analyses; 2. in model 2, adjustments were made for gender, type of admission, the APACHE IV score and the cumulative SOFA score during the entire ICU stay. Age is part of the APACHE IV score and was, therefore, not additionally entered in the model. By using the cumulative SOFA score, indirect correction for length of stay was made. Statistical significance was defined at a *P* value less than 0.05. The Statistical Package for Social Sciences 20.0 (SPSS 20.0) was used for all statistical analyses.

## Results

**Figure 1:**
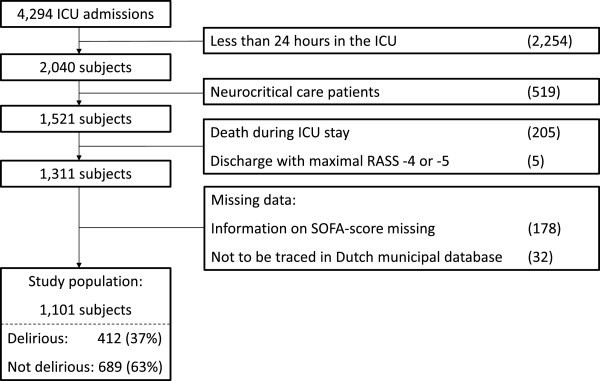
**Study flow chart.** ICU: Intensive Care Unit; RASS: Richmond Agitation Sedation Scale; SOFA: Sequential Organ Failure Assessment.

Between July 2009 and August 2011, 4,294 patients were admitted to the ICU, of whom 2,254 subjects stayed for less than one day. In addition, 519 neurocritical care patients were excluded. Of the remaining 1,521 subjects, 205 died during their ICU stay and 5 remained comatose during their ICU stay. Missing information about SOFA scores, or missing information in Dutch municipal database registration, resulted in the exclusion of another 210 subjects. Therefore, the final study population consisted of 1,101 persons, of whom 412 (37%) were delirious during their ICU stay (Figure 1). No patients were classified as delirious based on the prescription of haloperidol alone. After hospital discharge, 650 (59%) of the subjects could return home.

Characteristics of the study population are outlined in Table 1. Subjects who were excluded based on missing data did not differ from the study population with regard to delirium frequency, age, gender, APACHE IV or type of admission. The length of stay of the study population was longer than that of the subjects excluded due to missing data (median 4.0, IQR 3.0 to 7.0, *P* = 0.01).

**Table 1:** ICU characteristics of the study population

**Variables**	**All patients (n = 1,101)**	**No delirium (n = 689)**	**Delirium (n = 412)**	***P*-value**
Age (mean years, SD)	59.8 (16.5)	59.4 (16.6)	60.5 (16.7)	0.29
Male (number, %)	677 (61.5)	406 (58.9)	271 (65.8)	0.03
APACHE IV score (mean, SD)	61.3 (29.2)	53.9 (22.4)	73.7 (28.3)	<0.001
CumSOFA score (median, IQR)	40.0 (19.0 to 99.0)	27.0 (13.0 to 50.5)	99.0 (47.0 to 207.0)	<0.001
ICU length of stay (median days, IQR)	4.0 (3.0 to 8.0)	3.0 (2.0 to 5.0)	8.0 (5.0 to 15.0)	<0.001
Type of admission				
Medical (number, %)	430 (39.1)	208 (30.2)	222 (53.9)	<0.001
Elective surgical (number, %)	447 (40.6)	351 (50.9)	96 (23.3)	
Acute surgical (number, %)	224 (20.3)	130 (18.9)	94 (22.8)	

APACHE IV: Acute Physiology and Chronic Health Evaluation IV; CumSOFA: cumulative Sequential Organ Failure Assessment without the central nervous system component; IQR: interquartile range; SD: standard deviation*.*

Persons who were delirious during their ICU stay differed from subjects who did not have delirium during their ICU stay in gender, severity of illness scores, ICU length of stay and type of admission (Table 1). One year after ICU admission, 903 of the 1,101 subjects (82%) were known to have survived. Because of an administrative error, 16 individuals who appeared still alive did not receive a questionnaire, and, therefore, 887 surveys were sent. The response rate was 64% (571/887). After ICU admission, the median time until the surveys were sent back was 420 days after discharge (IQR 402 to 444 days).

In total, 198 patients died during follow-up, with a median duration of 62 days after ICU admission (IQR 25 to 181 days). Univariate survival analysis showed that delirious patients had a significantly increased risk of death in the year following ICU admission. However, when adjustments were made for the confounders described above, delirium was no longer independently associated with mortality (Table 2).

**Table 2:** Risk of death associated with delirium in survivors of critical illness, within one year after ICU admission

**Model**	**Hazard ratio, 95% CI**	***P*-value**
Crude	1.91 (1.44 to 2.52)	<0.001
Adjusted for gender, APACHE IV, type of admission and CumSOFA	1.26 (0.93 to 1.71)	0.14

198 patients died within one year. Delirious: n = 102, not delirious: n = 96. APACHE IV: Acute Physiology and Chronic Health Evaluation IV; CI: confidence interval; CumSOFA: cumulative Sequential Organ Failure Assessment without central nervous system component; ICU*:* intensive care unit.

In univariate analysis, patients with delirium during their ICU stay had a significantly lower HRQoL score at follow-up than patients who did not have delirium. After adjustment for confounders, again the difference between the two groups was no longer statistically significant (Table 3). The assumption of homoscedasticity was verified by plotting the residuals against the fitted values. Compared to the general Dutch population, both patient groups scored lower on the EQ-5D^TM^. Persons without delirium in the ICU scored 0.85 (IQR 0.72 to 1.00) and subjects with ICU delirium scored 0.75 (IQR 0.69 to 1.00). In comparison, the estimated average EQ-5D^TM^ index for the general Dutch population is 0.87 (IQR 0.82 to 1.00) [24].

**Table 3:** Differences in health-related quality of life between delirious and non-delirious ICU survivors, within one year after ICU admission

**Model**	**Difference, 95% CI**	***P*-value**
Crude	-0.06 (-0.10 to -0.01)	0.01
Adjusted for gender, APACHE IV, type of admission and CumSOFA	-0.04 (-0.10 to 0.01)	0.09

Data on health-related quality of life was available for 546 patients. Delirious: n = 182, not delirious: n = 364. APACHE IV: Acute Physiology and Chronic Health Evaluation IV; CI: confidence interval; CumSOFA: cumulative Sequential Organ Failure Assessment without central nervous system component; ICU: intensive care unit*.*

Persons who had been delirious during their ICU stay experienced significantly more mild and more severe self-reported problems in cognitive functioning compared to subjects who did not have delirium in the ICU. The strength of this association did not weaken and remained statistically significant when adjustments were made for confounding variables (Table 4).

**Table 4:** Risk of problems with cognitive functioning associated with delirium in survivors of critical illness, within one year after ICU admission

**Model**	**OR for mild problems with cognitive functioning, 95% CI**	***P*-value**	**OR for severe problems with cognitive functioning, 95% CI**	***P*-value**
Crude	2.02 (1.39 to 2.94)	<0.001	2.93 (1.16 to 7.42)	0.02
Adjusted for gender, APACHE IV, type of admission, and CumSOFA	2.41 (1.57 to 3.69)	<0.001	3.10 (1.10 to 8.74)	0.03

Data on cognitive functioning were available for 561 patients. The delirium group (n = 188) was divided into: No problems (n = 99), mild problems (n = 79) and severe problems (n = 10). For the group without delirium (n = 373): no problems (n = 261), mild problems (n = 103) and severe problems (n = 9). APACHE IV: Acute Physiology and Chronic Health Evaluation IV; CI: confidence interval; CumSOFA: cumulative Sequential Organ Failure Assessment without central nervous system component; ICU: intensive care unit; OR: odds ratio.

To verify whether the effect measure for mortality was robust when using the cumulative SOFA, we conducted sensitivity analyse where we made Cox proportional hazard models with the mean SOFA and the maximal SOFA scores. Furthermore, we evaluated the effect of adding length of stay to these models. Other variables were left unchanged. The HRs for death in these models remained similar, which shows that our effect measure is robust, and length of stay is not a mediator in the causal pathway between delirium and mortality.

## Discussion

We studied the association between delirium in the ICU and long-term mortality, HRQoL, and problems with cognitive functioning in survivors of critical illness. We found that delirium was not associated with mortality and HRQoL when adjustments were made for confounding. By contrast, subjects who had delirium during their ICU stay experienced more problems with cognitive functioning at follow-up than persons who did not have delirium in the ICU. The latter finding remained statistically significant when we adjusted for confounders, including estimates of severity of illness throughout the course of the ICU stay.

To the best of our knowledge, our study is the first to adjust for severity of illness throughout the course of the ICU stay, in analyzing the association between delirium with long-term mortality and HRQoL. Previous studies on these issues adjusted for severity of illness at baseline only [11-14,16]. Next to correction for severity of illness, differences with previous studies could be the result of differences in case mix, as we included ICU survivors only. Nevertheless, our study findings emphasize that the burden of illness during ICU stay should be taken into account. For example, a patient after elective surgery may have a low predicted mortality. However, when such a patient develops septic shock during their ICU stay, the risk of mortality may change but this is not incorporated in the APACHE IV score. Therefore, severity of illness at admission should not be considered the sole predictor of long-term outcome. To adjust for severity of illness throughout the course of the ICU stay, we used the cumulative SOFA score, which is dependent on both the duration and the extent of multi-organ failure, and which is strongly associated with long-term mortality [18]. We conducted sensitivity analyse with the mean SOFA and maximal SOFA, which showed that our effect measure was robust.

The association that we found between delirium in the ICU and long-term problems with cognitive functioning is consistent with a recent study, in which adjustments for severity of illness throughout the course of the ICU stay were made in a similar manner [10]. Factors that precipitate delirium may thus provoke events that contribute to the development or acceleration of cognitive impairment, even when delirium is no longer present. It would be interesting to see whether this holds only for persistent delirium or also for rapidly reversible, sedation-related delirium [25]. Unfortunately, we were not able to distinguish between these types of delirium.

The evidence of no association between delirium and long-term mortality and HRQoL should not be used as an excuse to neglect delirium in the ICU. With our study we show again that delirium is associated with prolonged cognitive problems [9-11]. Interventions aimed at reducing delirium incidence may eventually lead to long-term beneficial effects on cognitive outcome.

It is remarkable that the self-reported cognitive problems do not seem to have an impact on patients’ self-reported quality of life in this population. An association between more cognitive problems and a lower HRQoL would be expected. It might be due to a rather limited HRQoL survey. However, our findings are consistent with previous studies in which more extensive tools were used to assess HRQoL and cognitive functioning, namely the ShortForm 36 and the Cognitive Failure Questionnaire [11]. Perhaps the expectation to find a lower HRQoL in subjects with more cognitive problems is not always applicable.

No *a priori* sample size calculation was performed. However, this is one of the largest studies so far to address this problem. We believe that our study population was large enough to study this issue. Nevertheless, our study has several limitations. Due to missing data a relatively large group had to be excluded, which may have introduced bias. Excluded subjects had a shorter length of ICU stay than the study population and did not differ in other measured characteristics. Therefore, if selection bias would have occurred, we have analyzed a more severe group of subjects. Secondly, the sensitivity of the CAM-ICU in daily practice may be low [7]. Yet, in contrast to studies where sensitivity of the CAM-ICU was studied at one point in time, we used all CAM-ICU screenings during the patients’ entire ICU stay. As a result, we may have increased the sensitivity of the test, although this was not formally assessed [26]. We also used the prescription of haloperidol as a proxy measurement to reduce the risk of misclassification [20]. Thirdly, the duration of delirium as a measure for delirium burden is an important factor to incorporate in analyses [10], which was unfortunately not possible in our study. Fourthly, because of the lack of baseline assessment, it remains unclear from our data to what extent long-term cognitive problems are caused by delirium, and to what extent patients who experienced a delirium in the ICU had lower cognitive functioning before admission [17]. In a recent study on delirium and long-term cognitive impairment, this problem was addressed using the IQCODE for patients older than 50 years, excluding patients who were found to have severe dementia and stratifying according to age and burden of coexisting illness. This did not alter the observation that delirium increases the risk of long-term cognitive impairment [10]. Also, the response rate of the questionnaire was relatively low, which may have led to misclassification. Unfortunately, we were not able to address the issue of selective responsiveness. Furthermore, the assessment of problems with cognitive functioning with the EQ-6D is not detailed enough to examine specific functions or subdomains. The measure for cognitive functioning is based on a self-reported three-level multiple choice question, which is minimal compared to extensive neuropsychological testing. However, because of self-reporting, the test measures how subjects experience their cognitive functioning, which is a relevant outcome in daily practice. Finally, the possibility of bias due to unmeasured confounders cannot be excluded, as with any observational study.

## Conclusions

Delirium during ICU stay is not independently associated with long-term mortality and health related quality of life in ICU survivors when corrected for factors such as severity of illness throughout the course of the ICU stay. In contrast, delirium in the ICU increases the risk of long-term problems with cognitive functioning, independent of severity of illness during the ICU stay.

## Key messages

• In survivors of critical illness, delirium during their ICU stay is not related to mortality or long-term health related quality of life after adjusting for confounding, including severity of illness throughout the course of the ICU stay.

• ICU delirium appears to be an independent risk factor for self-reported long-term problems with cognitive functioning in ICU survivors.

## Abbreviations

APACHE IV: Acute Physiology and Chronic Health Evaluation IV; CAM-ICU: Confusion Assessment Method for the intensive care unit; CI: confidence interval; EQ-5D^TM^: European Quality of life – five dimensions; EQ-6D: European Quality of Life – six dimensions; HRQoL: Health Related Quality of Life; ICU: intensive care unit; IQR: interquartile range; RASS: Richmond Agitation and Sedation Scale; SD: standard deviation; SOFA: Sequential Organ Failure Assessment; SPSS 20.0: Statistical package for social sciences 20.0.

## Competing interests

The authors declare that they have no competing interests.

## Authors’ contributions

AEW contributed to the design of the study, carried out the main analyses, interpreted the data and wrote the manuscript. WP made a substantial contribution to the acquistion of the data by constructing the dataset, performed part of the analyses and reviewed the manuscript. OLC and DWL conceived the study, and participated substantially in its design and coordination and reviewed the manuscript critically. DD and AJCS participated substantially in the conception and design as well, helped in analyzing the data, and contributed to the draft and improvement of the manuscript. MFL contributed substantially to data interpretation and helped to draft and improve the manuscript. DSV helped substantially in analyzing the data and critically revised the manuscript for important intellectual content. All authors read and approved the final manuscript.

## References

[B1] Wolters AE Slooter AJC van der Kooi AW van Dijk D Cognitive impairment after intensive care unit admission: a systematic review Intensive Care Med 2013 39 376 386 23328935 10.1007/s00134-012-2784-9

[B2] Davydow DS Hough CL Langa KM Iwashyna TJ Pre-sepsis depressive symptoms are associated with incident cognitive impairment in survivors of severe sepsis: a prospective cohort study of older Americans J Am Geriatr Soc 2012 60 2290 2296 23176643 10.1111/jgs.12001 PMC3521098

[B3] Iwashyna TJ Ely EW Smith DM Langa KM Long-term cognitive impairment and functional disability among survivors of severe sepsis JAMA 2010 304 1787 1794 20978258 10.1001/jama.2010.1553 PMC3345288

[B4] Herridge MS Tansey CM Matté A Tomlinson G Diaz-Granados N Cooper A Guest CB Mazer CD Mehta S Stewart TE Kudlow P Cook D Slutsky AS Cheung AM Functional disability 5 years after acute respiratory distress syndrome N Engl J Med 2011 364 1293 1304 21470008 10.1056/NEJMoa1011802

[B5] Bienvenu OJ Colantuoni E Mendez-Tellez PA Dinglas VD Shanholtz C Husain N Dennison CR Herridge MS Pronovost PJ Needham DM Depressive symptoms and impaired physical function after acute lung injury Am J Respir Crit Care Med 2012 185 517 524 22161158 10.1164/rccm.201103-0503OC PMC3297105

[B6] American Psychiatry Association Diagnostic and Statistical Manual of Mental Disorders (DSM-IV-TR) 2000 4 Revision Washington, DC: American Psychiatric Association 135 147

[B7] Van Eijk MM van den Boogaard M van Marum RJ Benner P Eikelenboom P Honing ML van der Hoven B Horn J Izaks GJ Kalf A Karakus A Klijn IA Kuiper MA de Leeuw FE de Man T van der Mast RC Osse RJ de Rooij SE Spronk PE van der Voort PH van Gool WA Slooter AJ Routine use of the confusion assessment method for the intensive care unit: a multicenter study Am J Respir Crit Care Med 2011 184 340 344 21562131 10.1164/rccm.201101-0065OC

[B8] Pandharipande P Cotton BA Shintan A Thompson J Truman B Morris JA Dittus R Ely EW Prevalence and risk factors for development of delirium in surgical and trauma ICU patients J Trauma 2008 65 34 41 18580517 10.1097/TA.0b013e31814b2c4d PMC3773485

[B9] Girard TD Jackson JC Pandharipande PP Pun BT Thompson JL Shintani AK Gordon SM Canonico AE Dittus RS Bernard GR Ely EW Delirium as a predictor of long-term cognitive impairment in survivors of critical illness Crit Care Med 2010 38 1513 1520 20473145 10.1097/CCM.0b013e3181e47be1 PMC3638813

[B10] Pandharipande PP Girard TD Jackson JC Morandi A Thompson JL Pun BT Brummel NE Hughes CG Vasilevskis EE Shintani AK Moons KG Geevarghese SK Canonico AE Hopkins RO Bernard GR Dittus RS Ely EW Long-term cognitive impairment after critical illness N Engl J Med 2013 369 1306 1316 24088092 10.1056/NEJMoa1301372 PMC3922401

[B11] Van den Boogaard M Schoonhoven L Evers AW van der Hoeven JG van Achterberg T Pickkers P Delirium in critically ill patients: impact on long-term health-related quality of life and cognitive functioning Crit Care Med 2012 40 112 118 21926597 10.1097/CCM.0b013e31822e9fc9

[B12] Van Rompaey B Schuurmans MJ Shortridge-Baggett LM Truijen S Elseviers M Bossaert L Long term outcome after delirium in the intensive care unit J Clin Nurs 2009 18 3349 3357 19735334 10.1111/j.1365-2702.2009.02933.x

[B13] Ely EW Shintani A Truman B Speroff T Gordon SM Harrell FE Inouye SK Bernard GR Dittus RS Delirium as a predictor of mortality in mechanically ventilated patients in the intensive care unit JAMA 2004 291 1753 1762 15082703 10.1001/jama.291.14.1753

[B14] Abelha FJ Luís C Veiga D Parente D Fernandes V Santos P Botelho M Santos A Santos C Outcome and quality of life in patients with postoperative delirium during an ICU stay following major surgery Crit Care 2013 17 R257 24168808 10.1186/cc13084 PMC4057091

[B15] Gusi N Olivares PR Rajendram R The EQ-5D Health-Related Quality of Life Questionnaire Handbook of Disease Burdens and Quality of Life Measures 2010 New York: Springer 87 99

[B16] Svenningsen H Tønnesen EK Videbech P Frydenberg M Christensen D Egerod I Intensive care delirium - effect on memories and health-related quality of life - a follow-up study J Clin Nurs 2013 23 634 644 23647511 10.1111/jocn.12250

[B17] Slooter AJ Neurocritical care: critical illness, delirium and cognitive impairment Nat Rev Neurol 2013 9 666 667 24275929 10.1038/nrneurol.2013.235

[B18] Lone NI Walsh TS Impact of intensive care unit organ failures on mortality during the five years after a critical illness Am J Respir Crit Care Med 2012 186 640 647 22837381 10.1164/rccm.201201-0059OC

[B19] Ely EW Margolin R Francis J May L Truman B Dittus R Speroff T Gautam S Bernard GR Inouye SK Evaluation of delirium in critically ill patients: validation of the Confusion Assessment Method for the Intensive Care Unit (CAM-ICU) Crit Care Med 2001 29 1370 1379 11445689 10.1097/00003246-200107000-00012

[B20] Zaal IJ Spruyt CF Peelen LM van Eijk MM Wientjes R Schneider MM Kesecioglu J Slooter AJ Intensive care unit environment may affect the course of delirium Intensive Care Med 2013 39 481 488 23093246 10.1007/s00134-012-2726-6

[B21] Hoeymans N Sprangers MA Kwaliteit van leven gemeten met de EQ-6D. [The gateway to information about public health and healthcare.] [ http://www.nationaalkompas.nl/gezondheid-en-ziekte/functioneren-en-kwaliteit-van-leven/kwaliteit-van-leven/kwaliteit-van-leven-gemeten-met-de-eq-6d/]

[B22] Lamers LM Mcdonnell J Stalmeier PF Krabbe PF Busschbach JJV The Dutch tariff: results and arguments for an effective design for national EQ-5D valuation studies Health Econ 2006 15 1121 1132 16786549 10.1002/hec.1124

[B23] Szende A Oppe M Devlin N EQ-5D value sets; inventory, comparative review and user guide 2007 Dordrecht: Springer

[B24] Stolk E Krabbe P Busschbach J Using the Internet to collect EQ-5D norm scores: a valid alternative Proceedings of the 24th Scientific Plenary Meeting of the EuroQoL Group 2009 Kijkduin – The Hague: EuroQol Group 153 156

[B25] Patel SB Poston JT Pohlman A Hall JB Kress JP Rapidly reversible, sedation-related delirium versus persistent delirium in the intensive care unit Am J Respir Crit Care Med 2014 189 658 665 24423152 10.1164/rccm.201310-1815OC

[B26] Van den Boogaard M Pickkers P Slooter AJ Kuiper MA Spronk PE van der Voort PH van der Hoeven JG Donders R van Achterberg T Schoonhoven L Development and validation of PRE-DELIRIC ( PREdiction of DELIRium in ICu patients ) delirium prediction model for intensive care patients: observational multicentre study BMJ 2012 344 e420 22323509 10.1136/bmj.e420 PMC3276486

